# ﻿A new *Boulenophrys* species (Anura, Megophryidae) from the coastal hills of eastern Fujian Province, China

**DOI:** 10.3897/zookeys.1216.130017

**Published:** 2024-10-18

**Authors:** Shi-Shi Lin, Hong-Hui Chen, Yuan-Hang Li, Zhao-Ning Peng, Zhao-Chi Zeng, Jian Wang

**Affiliations:** 1 Guangdong Polytechnic of Environmental Protection Engineering, Foshan 528216, China Guangdong Polytechnic of Environmental Protection Engineering Foshan China

**Keywords:** *Boulenophryslichun* sp. nov., conservation actions, distribution pattern, diversity, Horned Toads, identification, new species, provincial key, taxonomy

## Abstract

A new species of the genus *Boulenophrys* is described from the coastal hills of eastern Fujian Province, China. The new taxon can be distinguished from all recognized congeners by a combination of discrete morphological character state differences and genetic divergences in the combined mitochondrial 16S + CO1 genes. We also provide a map showing the distribution pattern of *Boulenophrys* species in Fujian and a provincial-specific key, which will aid their conservation by helping the local authorities accurately identify species during field identifications and data collection efforts.

## ﻿Introduction

The Chinese Horned Toads (*Boulenophrys* Fei, Ye & Jiang, 2016) comprise 68 recognized species which are classified within the subfamily Megophryinae (Bonaparte, 1850) (Lyu et al. 2023; Zeng et al. 2024; Wang et al. 2024). They are widespread in the subtropical and tropical areas of mainland East Asia, mostly in southern China and southwards into northernmost Indochina, including Vietnam, Laos, Myanmar, and Thailand (Fei and Ye 2016; Lyu et al. 2023; Frost 2024). Located in southeastern China, Fujian Province possesses a complex mountain system. Its *Boulenophrys* diversity is still underestimated with three of the five species known from the area only described in recent years (Messenger et al. 2019; Lyu et al. 2021). Inger and Romer (1961) included a paratype of *B.brachykolos* (Inger & Romer, 1961) from Fujian, which may be a misidentification of *B.ombrophila* (Messenger & Dahn, 2019) due to morphological similarity (Lyu et al. 2023). Lyu et al. (2023) also restricted *B.brachykolos* to Hong Kong and Shenzhen in the east of the Pearl River Estuary based on voucher specimens and molecular data. Thus, only five recognized species occur in Fujian, namely *B.boettgeri* (Boulenger, 1899), *B.daiyunensis* (Lyu, Wang & Wang, 2021), *B.kuatunensis* (Pope, 1929), *B.ombrophila*, and *B.sanmingensis* (Lyu & Wang, 2021).

**Figure 1.**
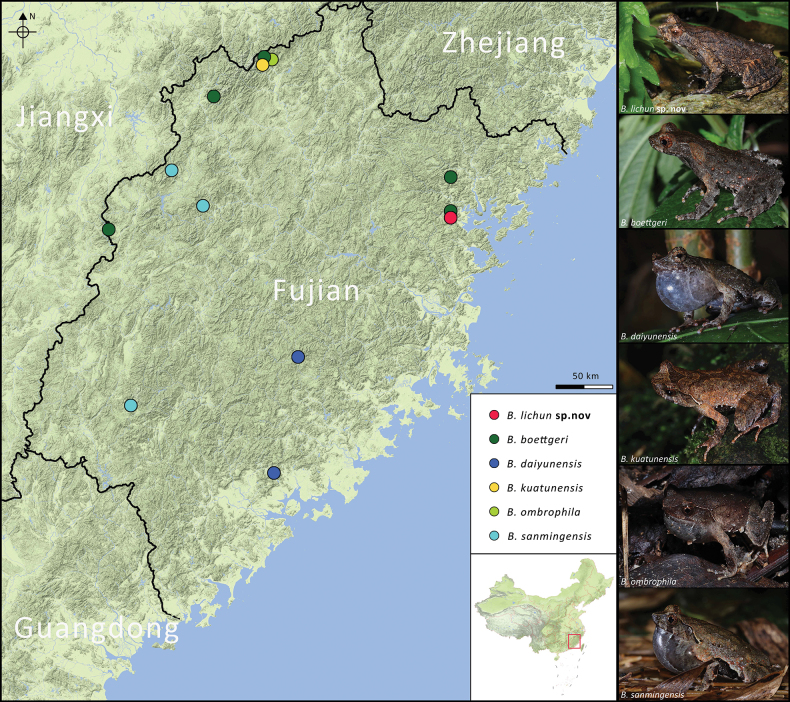
Map showing the distribution pattern of *Boulenophrys* species in Fujian Province, China. Distribution sites accessed from Lyu et al. (2023).

During recent field surveys in eastern Fujian, we collected a series of *Boulenophrys* specimens (Fig. 1). Preliminary morphological examination indicated that they could be distinguished from recognized congeners by a series of discrete characters. Subsequent molecular analysis further revealed that these specimens represent a separate evolutionary lineage, displaying significant divergence from known congeners. Thus, we describe them as a new species below.

## ﻿Materials and methods

### ﻿Morphology

All examined specimens were fixed in 10% buffered formalin and later transferred to 70% ethanol. All studied specimens have been deposited at the Guangdong Polytechnic of Environmental Protection Engineering (GEP), Foshan City, Guangdong, and the Herpetological Museum, Chengdu Institute of Biology, the Chinese Academy of Sciences (CIB), Chengdu City, China.

External measurements were recorded with a digital caliper (Neiko 01407A stainless steel 6-inch digital caliper) to the nearest 0.1 mm. These measurements are as follows: SVL (snout–vent length, from tip of snout to posterior margin of vent); HDL (head length, from tip of snout to the articulation of the jaw); HDW (head width, head width at the commissure of the jaws); SNT (snout length, from tip of snout to the anterior corner of the eye); IND (internasal distance, distance between nares); IOD (interorbital distance, minimum distance between upper eyelids); ED (eye diameter, from the anterior corner of the eye to posterior corner of the eye); TD (tympanum diameter, horizontal diameter of tympanum); TED (tympanum–eye distance, from anterior edge of tympanum to posterior corner of the eye); HND (hand length, from the proximal border of the outer palmar tubercle to the tip of digit III); RAD (forearm or radiulna length, from the flexed elbow to the proximal border of the outer palmar tubercle); FTL(foot length, from distal end of shank to the tip of digit IV); TIB (crus or tibiofibula length, from the outer surface of the flexed knee to the heel). Sex was determined by external secondary sexual characters, such as the presence of vocal sacs, nuptial pads, or spines in males and their absence in females (Fei et al. 2009).

Morphological characters of all 68 recognized species of the genus *Boulenophrys* used for comparisons were based on information available in the literature (Table 1).

**Table 1.** Literature for morphological characters of 68 recognized species of *Boulenophrys*.

*Boulenophrys* species	References
*B.acuta* (Wang, Li & Jin, 2014)	Li et al. 2014; Lyu et al. 2023
*B.angka* (Wu, Suwannapoom, Poyarkov, Pawangkhanant, Xu, Jin, Murphy & Che, 2019)	Wu et al. 2019
*B.anlongensis* (Li, Lu, Liu & Wang, 2020)	Li et al. 2020; Lyu et al. 2023
*B.baishanzuensis* (Wu, Li, Liu, Wang & Wu, 2020)	Wu et al. 2020
*B.baolongensis* (Ye, Fei & Xie, 2007)	Ye et al. 2007; Fei and Ye 2016
*B.binchuanensis* (Ye & Fei, 1995)	Lyu et al. 2023
*B.binlingensis* (Jiang, Fei & Ye, 2009)	Fei et al. 2009; Lyu et al. 2023
*B.boettgeri* (Boulenger, 1899)	Lyu et al. 2023
*B.brachykolos* (Inger & Romer, 1961)	Lyu et al. 2023
*B.caobangensis* (Nguyen, Pham, Nguyen, Luong & Ziegler, 2020)	Nguyen et al. 2020
*B.caudoprocta* (Shen, 1994)	Shen 1994; Lyu et al. 2023
*B.congjiangensis* (Luo, Wang, Wang, Lu, Wang, Deng & Zhou, 2021)	Luo et al. 2021; Lyu et al. 2023
*B.cheni* (Wang & Liu, 2014)	Wang et al. 2014; Lyu et al. 2023
*B.chishuiensis* (Xu, Li, Liu, Wei & Wang, 2020)	Lyu et al. 2023
*B.daiyunensis* (Lyu, Wang & Wang, 2021)	Lyu et al. 2021; Lyu et al. 2023
*B.daoji* (Lyu, Zeng, Wang & Wang, 2021)	Lyu et al. 2021; Lyu et al. 2023
*B.daweimontis* (Rao & Yang, 1997)	Rao and Yang 1997
*B.dongguanensis* (Wang & Wang, 2019)	Lyu et al. 2023
*B.elongata* Zeng, Wang, Chen, Xiao, Zhan, Li & Lin, 2024	Zeng et al. 2024
*B.fengshunensis* Wang, Zeng, Lyu & Wang, 2022	Lyu et al. 2023
*B.fanjingmontis* (Zhang, Liang, Ran & Shen, 2012)	Lyu et al. 2023
*B.fansipanensis* (Tapley, Cutajar, Mahony, Nguyen, Dau, Luong, Le, Nguyen, Nguyen, Portway, Luong & Rowley, 2018)	Tapley et al. 2018a
*B.frigida* (Tapley, Cutaja, Nguyen, Portway, Mahony, Nguyen, Harding, Luong & Rowley, 2021)	Tapley et al. 2021
*B.hoanglienensis* (Tapley, Cutajar, Mahony, Nguyen, Dau, Luong, Le, Nguyen, Nguyen, Portway, Luong & Rowley, 2018)	Tapley et al. 2018a
*B.hungtai* Wang, Zeng, Lyu, Xiao & Wang, 2022	Lyu et al. 2023
*B.hengshanensis* Qian, Hu, Mo, Gao, Zhang & Yang, 2023	Qian et al. 2023
*B.insularis* (Wang, Liu, Lyu, Zeng & Wang, 2017)	Wang et al. 2017a; Lyu et al. 2023
*B.jiangi* (Liu, Li, Wei, Xu, Cheng, Wang & Wu, 2020)	Lyu et al. 2023
*B.jingdongensis* (Fei & Ye, 1983)	Fei et al. 1983; Lyu et al. 2023
*B.jinggangensis* (Wang, 2012)	Wang et al. 2012; Lyu et al. 2023
*B.jiulianensis* (Wang, Zeng, Lyu & Wang, 2019)	Lyu et al. 2023
*B.kuatunensis* (Pope, 1929)	Lyu et al. 2023
*B.leishanensis* (Li, Xu, Liu, Jiang, Wei & Wang, 2018)	Lyu et al. 2023
*B.lushuiensis* (Shi, Li, Zhu, Jiang, Jiang & Wang, 2021)	Wang et al. 2017b; Lyu et al. 2023
*B.liboensis* (Zhang, Li, Xiao, Li, Pan, Wang, Zhang & Zhou, 2017)	Zhang et al. 2017
*B.lini* (Wang & Yang, 2014)	Wang et al. 2014; Lyu et al. 2023
*B.lishuiensis* (Wang, Liu & Jiang, 2017)	Lyu et al. 2023
*B.minor* (Stejneger, 1926)	Lyu et al. 2023
*B.mirabilis* (Lyu, Wang & Zhao, 2020)	Lyu et al. 2020; Lyu et al. 2023
*B.mufumontana* (Wang, Lyu & Wang, 2019)	Lyu et al. 2023
*B.nankunensis* (Wang, Zeng & Wang, 2019)	Lyu et al. 2023
*B.nanlingensis* (Lyu, Wang, Liu & Wang, 2019)	Lyu et al. 2023
*B.obesa* (Wang, Li & Zhao, 2014)	Li et al. 2014; Lyu et al. 2023
*B.ombrophila* (Messenger & Dahn, 2019)	Lyu et al. 2023
*B.omeimontis* (Liu, 1950)	Lyu et al. 2023
*B.palpebralespinosa* (Bourret, 1937)	Fei et al. 2009; Lyu et al. 2023
*B.pepe* (Wang & Zeng, 2024)	Wang et al. 2024
*B.puningensis* Wang, Zeng, Lyu, Xiao & Wang, 2022	Lyu et al. 2023
*B.qianbeinsis* (Su, Shi, Wu, Li, Yao, Wang & Li, 2020)	Lyu et al. 2023
*B.rubrimera* (Tapley, Cutajar, Mahony, Chung, Dau, Nguyen, Luong & Rowley, 2017)	Tapley et al. 2017, 2018b
*B.sangzhiensis* (Jiang, Ye & Fei, 2008)	Lyu et al. 2023
*B.sanmingensis* (Lyu & Wang, 2021)	Lyu et al. 2021; Lyu et al. 2023
*B.shimentaina* (Lyu, Liu & Wang, 2020)	Lyu et al. 2020; Lyu et al. 2023
*B.shuichengensis* (Tian & Sun, 1995)	Tian and Sun 1995; Tian et al. 2000; Fei and Ye 2016
*B.shunhuangensis* (Wang, Deng, Liu, Wu & Liu, 2019)	Wang et al. 2019b; Lyu et al. 2023
*B.spinata* (Liu & Hu, 1973)	Hu et al. 1973; Lyu et al. 2023
*B.tongboensis* (Wang & Lyu, 2021)	Lyu et al. 2021; Lyu et al. 2023
*B.tuberogranulatus* (Shen, Mo & Li, 2010)	Mo et al. 2010; Fei and Ye 2016; Lyu et al. 2023
*B.wugongensis* (Wang, Lyu & Wang, 2019)	Lyu et al. 2023
*B.wuliangshanensis* (Ye & Fei, 1995)	Lyu et al. 2023
*B.wushanensis* (Ye & Fei, 1995)	Ye and Fei 1995; Fei and Ye 2016; Lyu et al. 2023
*B.xiangnanensis* (Lyu, Zeng & Wang, 2020)	Lyu et al. 2020; Lyu et al. 2023
*B.xianjuensis* (Wang, Wu, Peng, Shi, Lu & Wu, 2020)	Lyu et al. 2023
*B.xuefengmontis* Lyu & Wang, 2023	Lyu et al. 2023
*B.yangmingensis* (Lyu, Zeng & Wang, 2020)	Lyu et al. 2020; Lyu et al. 2023
*B.yaoshanensis* Qi, Mo, Lyu, Wang & Wang, 2021	Qi et al. 2021; Lyu et al. 2023
*B.yingdeensis* Qi, Lyu, Wang & Wang, 2021	Qi et al. 2021; Lyu et al. 2023
*B.yunkaiensis* Qi, Wang, Lyu & Wang, 2021	Qi et al. 2021; Lyu et al. 2023

### ﻿Phylogeny

We use two partial mitochondrial genes, the 16S ribosomal RNA (16S) and the cytochrome *c* oxidase 1 (COI), for phylogenetic analysis. DNA extraction, PCR amplification, and sequencing protocols follow that of Liu et al. (2018). In total, 84 sequences were used in this study, including six new ones from this study and 78 attained from GenBank. Two samples of the genus *Xenophrys* were used as outgroups (Suppl. material 1).

We used Clustal X 2.0 (Thompson et al. 1997) for sequence aligning with default parameters. PartitionFinder (Lanfear et al. 2012) was used for searching the optimal partitioning schemes and the analysis determined that partitioning by gene was optimal for 16S, while partitioning by codon position was optimal for COI, with GTR+I+G identified as the best-fit nucleotide substitution model for all partitions. Phylogenetic trees were constructed using maximum likelihood (ML) implemented in RaxmlGUI v.1.3 (Silvestro and Michalak 2012), and Bayesian inference (BI) using MrBayes v.3.2.4 (Ronquist et al. 2012). For the ML analysis, an optimal tree was obtained and branch supports were evaluated with 1000 rapid bootstrapping replicates. For the BI analysis, two independent runs were conducted with each running for 10,000,000 generations and sampled every 1000 generations with the first 25% samples discarded as burn-in, resulting in a potential scale reduction factor (PSRF) of < 0.01. Then the remaining trees were used to create a consensus tree. Nodes having ML bootstrap values (BS) ≥ 70 and BI posterior probabilities (BPP) ≥ 0.90 were considered well supported.

## ﻿Results

**Figure 2.**
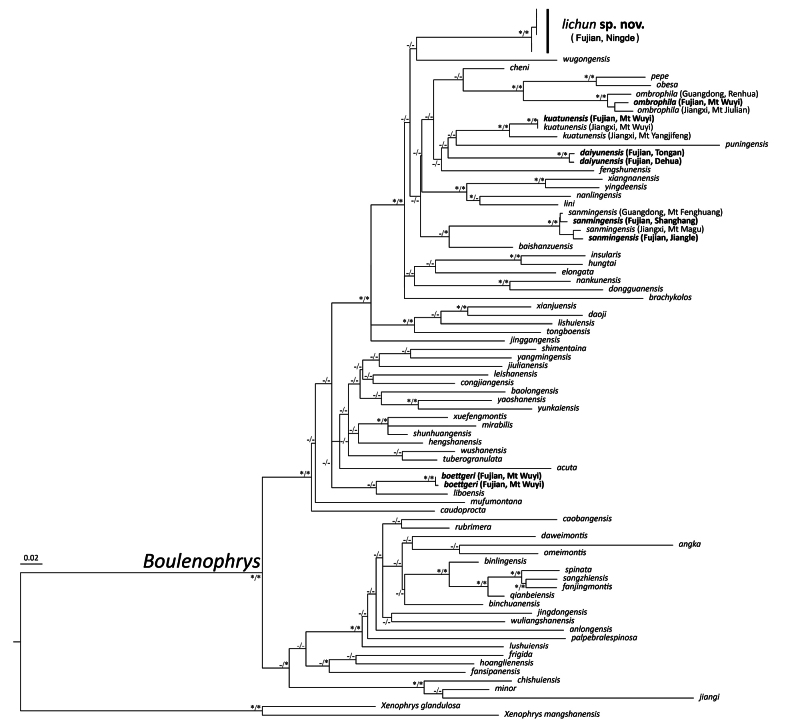
Maximum-likelihood phylogenies, with ‘*’ representing BS ≥ 70 or BPP ≥ 0.90 and ‘-’ representing BS < 70 or BPP < 0.90.

The BI and ML phylogenetic trees resulted in essentially identical topologies, with the ML phylogenetic tree shown in Fig. 2. The relationship among the *Boulenophrys* species in our trees correspond to those in previous studies (Liu et al. 2018; Lyu et al. 2023; Zeng et al. 2024; Wang et al. 2024). Our results show that all samples from eastern Fujian, China, cluster into a monophyletic group with strong nodal support (BS 100, BPP 1.00), and are distinct from all known *Boulenophrys* species occurring in Fujian. Furthermore, the corresponding specimens of the evolving lineage can be distinguished from all recognized congeners by a combination of morphological characters. As both the phylogenetic results and morphological comparisons support that the lineage from eastern Fujian represents an undescribed new species, we thus describe it below.

### ﻿Taxonomic account

#### ﻿ Boulenophrys lichun sp. nov.

Taxon classificationAnimaliaAnuraMegophryidae

AF6296E2-B161-5E10-8192-74EAB15B0D8F

https://zoobank.org/02303B1E-ED1A-482B-8BD3-B32BC0DB9D6B

Fig. 3

##### Material examined.

***Holotype*.** China • ♂; Fujian Province, Ningde City, Jiaocheng District, Mt Nanji; 26.645774°N, 119.519939°E, ca. 230 m elev.; 4 Feb. 2024; Jian Wang, Zhao-Chi Zeng, Shi-Shi Lin and Yuan-Hang Li leg.; GEP a214.

**Figure 3.**
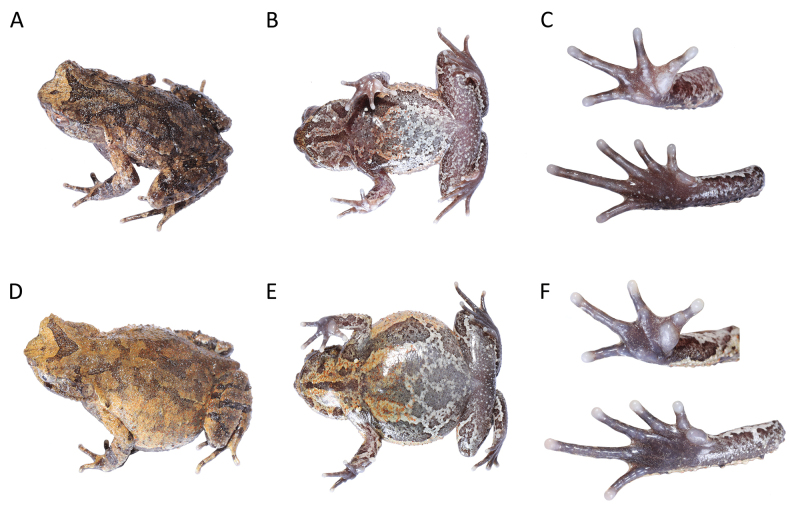
The male holotype (GEP a214, **A–C**) and the female paratype (GEP a215, **D–F**) of *Boulenophryslichun* sp. nov. in life.

***Paratypes*.** China • 4♂♂; same data as for holotype; CIB 121428 [field number GEP a210], GEP a211–213 • 1♀; same data as for holotype; GEP a215.

##### Etymology.

The specific name *lichun* is derived from Chinese Pinyin Lì Chūn, i.e. 立春 in Chinese, which means the beginning of spring, the first of the 24 solar terms (24节气) of China. The specific name refers to the breeding season of the new species which begins around this period. The song of the new species heralds the spring of a year. The type specimens of the new species were also collected on “Lichun” of the Year 2024.

##### Diagnosis.

(1) small size (SVL 33.5–37.0 mm in five adult males, SVL 47.1 mm in a single adult female); (2) canthus rostralis well developed, tongue not notched posteriorly; (3) tympanum distinct; (4) vomerine ridges and vomerine teeth present; (5) dorsal skin rough and highly granular, discontinuous X-shaped ridge on center of dorsum, discontinuous dorsolateral ridges present, sparse large tubercles on flanks, dorsal limbs with discontinuous transverse ridges and tubercles, ventral skin with dense raised tubercles; (6) outer margin of upper eyelid with a small horn-like prominent tubercle, supratympanic fold distinct and narrow, curving posteroventrally to above arm; (7) two metacarpal tubercles distinct, inner one observably enlarged; relative finger lengths I < II < IV < III; distinct subarticular tubercle at base of each finger; (8) heels not meeting when hindlimbs folded; tibio-tarsal articulation reaching shoulder to posterior corner of eye; (9) toes without webbing and lateral fringes, inner metatarsal tubercle long ovoid, outer one absent, relative toe length I < II < V < III < IV; (10) dorsal surface yellowish-brown with irregular dark-brown patches, and dark-brown triangular marking between eyes, dorsal limbs and digits light brown with dark-brown transverse bands; and (11) dense nuptial spines on dorsal bases of fingers I and II in breeding adult males, subgular vocal sac present in males.

##### Description of holotype.

Adult male. Body size small, SVL 37.0 mm. Head width larger than head length, HWD/HDL 1.04; snout rounded in dorsal view, projecting, sloping backward to mouth in profile, protruding well beyond margin of lower jaw; top of head flat; eyes moderate in size, ED 0.34 of HDL, pupil vertical, near diamond-shaped; nostril obliquely ovoid; canthus rostralis well developed; loreal region slightly oblique; internasal distance slightly larger than interorbital distance; tympanic region oblique, tympanum distinct and visible in dorsal view; tympanum moderate in size, margin clear, upper margin in contact with supratympanic fold, lower margin in contact with upper lip, TD/ED 0.55; large ovoid choanae at base of maxilla; vomerine ridge and vomerine teeth present, maxillary teeth present; margin of tongue rounded, not notched distally; presence of single subgular vocal sac.

Forearm length 0.23 of SVL, hand 0.24 of SVL; webbing absent between fingers, lateral fringes absent, relative finger length I < II < IV < III; tips of fingers slightly dilated, round; subarticular tubercles on base of fingers present, distinct; inner metacarpal tubercle observably enlarged, outer one slightly smaller; single nuptial pad bearing nuptial spines present on dorsal surface of first and second fingers, respectively. Hindlimbs short, tibio-tarsal articulation reaching forward to posterior conner of eye when hindlimb stretched along body; heels not meeting when flexed hindlimbs held at right angles to body axis; crus length 0.40 of SVL and foot length 0.58 of SVL; relative toe length I < II < V < III < IV; tips of toes round and slightly dilated; toes without lateral fringes and webbing; subarticular tubercles on base of toes present and distinct; inner metatarsal tubercle long ovoid and lacking outer metatarsal tubercle.

Dorsal skin rough and highly granular; dense large tubercles on flanks; single horn-like prominent tubercle on edge of upper eyelid; obvious supratympanic fold curving posteroventrally from posterior corner of eye to level above insertion of arm; upper lip, mandibular articulation, loreal, temporal region excluding tympanum, upper eyelid and surface around cloaca with conical tubercles; discontinuous X-shaped ridge on center of dorsum, discontinuous dorsolateral ridges present; ventral surface with dense raised tubercles; tubercles on ventral hindlimbs and around cloaca bearing tiny spines on their tips; small and distinct pectoral gland closer to axilla; single femoral gland positioned on posterior surface of thigh at midpoint between knee and cloaca.

##### Coloration of holotype.

In life, dorsal surface of body yellowish-brown with irregular dark-brown patches, dark-brown X-shaped marking on center of dorsum, dark-brown triangular marking between eyes. A vertical dark-brown band present below eye. Dorsal surface of limbs with dark-brown transverse bands. Tubercles on edge of upper eyelids orange. Supratympanic fold light brown. Surface of throat and chest yellowish-brown with irregular dark brown and white patches and white and orange dots. Center of throat with black longitudinal band. Surface of abdomen white, mottled with orange dots and black patches. Surface of ventral limbs purple brown, with white mottling and dark-brown patches. Spines on tips of tubercles on ventral hindlimbs and area around cloaca black. Digits gray white; subarticular tubercles, inner and outer metacarpal tubercles and inner metatarsal tubercle grayish-brown. Pectoral glands and femoral glands white. Iris yellowish-brown with range mottling.

In preservative, the dorsal surface of the body is dark brown, with markings and patches more distinct. Surface of chest, throat and limbs are dark brown, with dark-brown markings and patches more distinct, white patches and dots faded and orange dots absent. Color of pectoral glands and femoral glands faded.

##### Variation.

Morphometric variation is listed in Table 2. Most of the paratypes are similar to the holotype in morphology and color pattern, except for the following: tibio-tarsal articulation reaching forward to posterior corner of eye when hindlimb stretched along body in the holotype GEP a214, while reaching to shoulder in female paratype GEP a215; absence of nuptial pads and spines in the female paratype; larger body size in the female paratype.

**Table 2.** Measurements (in mm) of voucher specimens of *Boulenophryslichun* sp. nov.; * holotype.

Voucher	CIB 121428	GEP a211	GEP a212	GEP a213	GEP a214 *	GEP a215
**Sex**	male	male	male	male	male	female
**SVL**	36.7	33.5	35.7	34.6	37.0	47.1
**HDL**	13.1	12.7	13.5	12.9	13.3	15.3
**HDW**	13.6	13.3	13.8	13.6	13.9	16.5
**ED**	4.5	4.0	4.5	4.5	4.8	5.5
**TD**	2.3	2.6	2.5	2.5	2.6	2.8
**TED**	1.7	1.7	1.8	1.8	1.7	2.4
**SNT**	4.5	4.4	4.5	4.5	4.6	4.9
**IND**	3.6	3.7	3.8	3.7	3.9	4.3
**IOD**	3.5	3.3	3.4	3.5	3.7	4.3
**HDN**	8.7	7.8	8.7	9.1	9.2	10.2
**RAD**	8.6	7.0	8.5	8.1	8.7	9.0
**FTL**	20.7	18.9	20.7	20.9	21.4	24.1
**TIB**	14.3	12.5	14.3	14.5	14.7	15.7

##### Comparisons.

*Boulenophryslichun* sp. nov. can easily be distinguished from the following congeners by its heels not meeting when flexed hindlimbs held at right angles to body axis: *B.anlongensis*, *B.baishanzuensis*, *B.binlingensis*, *B.caudoprocta*, *B.cheni*, *B.chishuiensis*, *B.congjiangensis*, *B.daweimontis*, *B.fanjingmontis*, *B.fansipanensis*, *B.frigida*, *B.hoanglienensis*, *B.jiangi*, *B.jingdongensis*, *B.jinggangensis*, *B.jiulianensis*, *B.leishanensis*, *B.liboensis*, *B.lini*, *B.lushuiensis*, *B mirabilis*, *B.mufumontana*, *B.nanlingensis*, *B.omeimontis*, *B.palpebralespinosa*, *B.qianbeiensis*, *B.sangzhiensis*, *B.sanmingensis*, *B.shimentaina*, *B.shunhuangensis*, *B.spinata*, *B.sanmingensis*, *B.tongboensis*, *B.tuberogranulatus*, *B.wuliangshanensis*, *B.xianjuensis*, *B.yangmingensis*, *B.yaoshanensis*, *B.yingdeensis* (vs. heels overlapping), from *B.binchuanensis*, *B.elongata*, *B.lishuiensis*, *B.minor*, *B.xiangnanensis*, *B.xuefengmontis* (vs. heels just meeting), and from *B.angka*, *B.daiyunensis*, *B.baolongensis*, *B.wushanensis*, *B.yunkaiensis* (vs. heels just meeting or slightly overlapping).

*Boulenophryslichun* sp. nov. can easily be distinguished from the following congeners by its tongue not notched distally: *B.brachykolos*, *B.insularis*, *B.pepe* (vs. tongue notched distally). *Boulenophryslichun* sp. nov. can easily be distinguished from the following congeners by its presence of vomerine teeth: *B.acuta*, *B.boettgeri*, *B.caobangensis*, *B.daoji*, *B.hungtai*, *B.hengshanensis*, *B.kuatunensis*, *B.ombrophila*, *B.obesa*, *B.shuichengensis*, *B.wugongensis* (vs. vomerine teeth absent).

*Boulenophryslichun* sp. nov. can easily be distinguished from the following congeners by its absence of lateral fringes on webbing on toes: *B.dongguanensis*, *B.fengshunensis*, *B.nankunensis*, *B.puningensis* (vs. toes with rudimentary webbing), and from *B.rubrimera* (vs. toes with narrow lateral fringes).

##### Distribution and natural history.

Currently, *Boulenophryslichun* sp. nov. is only known from the coastal hills of Ningde City, eastern Fujian Province, China. It inhabits flowing montane seeps and the nearby forest floor and leaf litter. The habitat is surrounded by secondary forest mixed with bamboo groves at elevations between 150–510 m. Advertisement calls of males were heard from February to May. Males were found calling in rock crevices.

## ﻿Discussion

The lack of follow-up surveys can pose issues in terms of endangered species listing. *Boulenophryslichun* is currently only known from the coastal hills of Ningde City, eastern Fujian. The development of tourism infrastructure, stream diversion and tea leaf cultivation have gradually affected and threatened the habitats of the new species. Thus, more data (i.e., distribution, population size, potential and existing risk factors, etc.) from long-term extensive surveys are urgently required to make an assessment of their endangered status.

*Boulenophrys* possess limited dispersal abilities and narrow ecological niches, resulting in the restricted distribution ranges of many species (Wang et al. 2019a; Lyu et al. 2023). Their high levels of morphological conservatism (Liu et al. 2018; Wang et al. 2022; Lyu et al. 2023) and the lack of follow-up surveys have led to inadequate protection due to misidentifications and deficient data. Thus, we provide a provincial key below to aid the local authorities in accurately identifying species during field identifications and data collection efforts and further serve their conservation.

### ﻿Key to *Boulenophrys* species occurring in Fujian Province, China

**Table:** 

1	Vomerine ridges and vomerine teeth present	**2**
–	Vomerine ridges and vomerine teeth absent	**3**
2	Toes with rudimentary webbing and wide lateral fringes	***B.daiyunensis***
–	Toes without webbing and lateral fringes	***B.lichun***
3	Toes with rudimentary webbing and lateral fringes	**4**
–	Toes without webbing and lateral fringes	***B.ombrophila***
4	Toes with wide lateral fringes	**5**
–	Toes with narrow lateral fringes	***B.kuatunensis***
5	Round light patches on the shoulder present	***B.boettgeri***
–	Round light patches on the shoulder absent	***B.sanmingensis***

## Supplementary Material

XML Treatment for Boulenophrys lichun

## ﻿Additional information

### Author ORCIDs

Shi-Shi Lin https://orcid.org/0000-0001-7709-7005

Hong-Hui Chen https://orcid.org/0000-0001-5026-2920

Yuan-Hang Li https://orcid.org/0009-0009-6543-9245

Zhao-Ning Peng https://orcid.org/0009-0008-5103-2797

Zhao-Chi Zeng https://orcid.org/0000-0003-4054-8399

Jian Wang https://orcid.org/0000-0003-4249-7767

### Data availability

All of the data that support the findings of this study are available in the main text or Supplementary Information.

## Supplementary materials

Supplementary material 1 Localities, voucher information, and GenBank accession numbers for all samples used in this study Data type xlsx https://binary.pensoft.net/file/1156128This dataset is made available under the Open Database License (http://opendatacommons.org/licenses/odbl/1.0/). The Open Database License (ODbL) is a license agreement intended to allow users to freely share, modify, and use this Dataset while maintaining this same freedom for others, provided that the original source and author(s) are credited.Shi-Shi Lin, Hong-Hui Chen, Yuan-Hang Li, Zhao-Ning Peng, Zhao-Chi Zeng, Jian Wang
